# Exploring Serum BDNF as a Biomarker in Negative Symptoms of Schizophrenia following Neuromodulatory Intervention

**DOI:** 10.1192/j.eurpsy.2026.10716

**Published:** 2026-07-31

**Authors:** R. Gupta, A. Sharma

**Affiliations:** 1 Psychiatry, Institute of Mental Health, Rohtak; 2 Psychiatry, Central Institute of Psychiatry, Ranchi, India

## Abstract

**Introduction:**

Schizophrenia with predominantly negative symptoms is highly disabling and lacks effective treatments. This gap underscores the need for biomarkers to guide novel approaches. Brain-Derived Neurotrophic Factor (BDNF), essential for neuronal survival and synaptic plasticity, is consistently reduced in schizophrenia, especially in those with prominent negative symptoms. Although its role as causative or epiphenomenal remains debated, its involvement in neurodevelopment and synaptic regulation makes it a strong candidate biomarker. Neuromodulatory interventions like rTMS and tDCS enhance neuroplasticity with emerging evidence in schizophrenia. The combination of HD-tDCS priming with iTBS offers a promising strategy targeting DLPFC hypoactivity, leveraging metaplasticity to potentiate BDNF-mediated plasticity. Investigating serum BDNF may provide insights into the biological underpinnings of neuromodulation & possibility of developing biomarker-guided precision psychiatry for schizophrenia.

**Objectives:**

1. To measure and compare serum BDNF levels before and after HD-tDCS (active/sham) priming followed by iTBS.

2. To examine the correlation between serum BDNF levels and clinical symptom severity, including PANSS and SANS scores.

**Methods:**

A prospective hospital based double-blinded randomized controlled study with a sample size of 40, where the participants were divided into Active or Sham HD-tDCS primed iTBS stimulation groups for a total of 20 sessions over two weeks. Serum BDNF was quantified at baseline and after 4 weeks using Sandwich ELISA kits. Symptom severity was assessed with PANSS and SANS. Data were analyzed using paired and independent t-tests, along with correlational analyses.

**Results:**

At baseline, serum BDNF did not differ significantly between active and sham groups. After 4 weeks, BDNF increased significantly in the active group, while a non-significant decline was observed in the sham group. Between-group comparison at 4 weeks favored the active group but did not reach statistical significance. Correlational analysis showed baseline serum BDNF negatively correlated with total PANSS scores. Post-treatment, higher serum BDNF levels correlated with lower PANSS total, PANSS negative subscale, and SANS domains including affective flattening and alogia. These findings suggest that increases in BDNF are linked to reductions in overall symptom severity.

**Conclusions:**

HD-tDCS primed iTBS significantly increased serum BDNF levels in patients with negative symptoms of schizophrenia, with higher BDNF associated with reduced clinical severity. While between-group differences were not statistically significant, the results suggest that BDNF may serve as a biomarker of neuromodulation-induced neuroplasticity. Future studies with larger cohorts and longer follow-up are required to validate these findings and explore prognostic applications.

**Disclosure of Interest:**

None Declared

